# Hypoxia-Induced Adipose Lipolysis Requires Fibroblast Growth Factor 21

**DOI:** 10.3389/fphar.2020.01279

**Published:** 2020-08-14

**Authors:** Guicheng Wu, Yanlong Liu, Wenke Feng, Xuan An, Wenhui Lin, Chengwei Tang

**Affiliations:** ^1^Department of Gastroenterology, West China Hospital, Sichuan University, Chengdu, China; ^2^Department of Hepatology, Chongqing University Three Gorges Hospital, Chongqing, China; ^3^School of Mental Health, Wenzhou Medical University, Wenzhou, China; ^4^Zhuji Institute of Biomedicine, School of Pharmaceutical Sciences, Wenzhou Medical University, Shaoxing, China; ^5^Alcohol Research Center, University of Louisville School of Medicine, Louisville, KY, United States; ^6^Department of Cardiology, Affiliated Wenling Hospital of Wenzhou Medical University, Wenling, China; ^7^Laboratory of Gastroenterology & Hepatology, State Key Laboratory of Biotherapy, Chengdu, China

**Keywords:** fibroblast growth factor 21, free fatty acid, lipolysis, hormone sensitive lipase, hypoxia-inducible factor

## Abstract

Fibroblast growth factor 21 (FGF21) is a recently discovered hepatokine that regulates lipid and glucose metabolism and is upregulated in response to numerous physiological and pathological stimuli. Herein, we demonstrate that both physical and chemical hypoxia increase the systemic and hepatic expression of FGF21 in mice; by contrast, hypoxia induces a reduction of FGF21 expression in hepatocytes, indicating that hypoxia-induced FGF21 expression is differentially regulated in intact animals and in hepatocytes. Furthermore, we demonstrate that hypoxia treatment increases hormone-sensitive lipase-mediated adipose tissue lipolysis in mice, which is reduced in *Fgf21* knockout mice, thereby implying that FGF21 plays a critical role in hypoxia-related adipose lipolysis. Adipose tissue lipolysis causes an increase in the amount of circulating free fatty acids, which leads to the activation of peroxisome proliferators-activated receptor alpha and an increased expression of FGF21 in hepatocytes. We further show that hypoxia-induced elevation of reactive oxygen species, but not the hypoxia-inducible factor, is responsible for the lipolysis and FGF21 expression. In conclusion, our data clearly demonstrate that FGF21 plays a critical role in hypoxia-induced adipose lipolysis, which induces hepatic expression of FGF21. Clarification of hypoxia-regulated FGF21 regulation will enhance our understanding of the pathophysiology of hypoxia-related diseases, such as sleep disorders and metabolic diseases.

## Introduction

Adipose tissue functions as the major fat storage site in the form of triglycerides (TGs). Adipose tissue synthesizes TG when energy intake exceeds energy output, while during fasting or in response to stress, adipose tissue mobilizes free fatty acids (FFAs) and glycerol (lipolysis), providing other tissues with metabolites and energy substrates. While lipolysis is a physiological response to metabolic changes, excess lipolysis may lead to increased circulating FFA levels, which is a risk factor for a variety of metabolic diseases. Previous studies have shown that adipose tissue lipolysis is associated with tissue hypoxia (Mahat et al., 2016), which is a pathophysiological state existing in many disease conditions (Famulla et al., 2012; Plihalova et al., 2016; Zhang et al., 2017). At molecular level, lipolysis and hypoxia have been linked to fibroblast growth factor 21 (FGF21), respectively (Arner et al., 2008; Cai et al., 2019).

FGF21 is a member of the FGF superfamily and is a potential metabolic regulator (Degirolamo et al., 2016; Kliewer and Mangelsdorf, 2019; Lewis et al., 2019). Generally, FGFs require heparin to promote binding to the tyrosine kinase FGF receptors (FGFRs) and act in a paracrine or autocrine fashion (Beenken and Mohammadi, 2009). FGF21 does not possess a heparin-binding domain and is therefore secreted into the circulation, where it exerts its functions through the activation of a unique dual receptor system consisting of FGFRs and a co-receptor, β-klotho (Ding et al., 2012); in this way, it can function in an endocrine fashion.

Circulating FGF21 levels have been found to be elevated in patients and experimental animals with various metabolic conditions, such as obesity (Dushay et al., 2010), non-alcoholic fatty liver disease (Kim et al., 2015), and type 2 diabetes (Barb et al., 2019). Besides, previous studies have demonstrated that there was a association between hypoxia and FGF21 regulation in many diseases such as type 2 diabetes with early renal injury (Yu et al., 2019), pulmonary hypertension (Cai et al., 2019), and cerebral disease (Wang et al., 2019). Hypoxic conditions increase the circulating concentrations of FFAs, which are produced from adipose tissue by increased lipolysis (Yin et al., 2009). The FFAs, in turn, activate peroxisome proliferators-activated receptor alpha (PPARα) and increase FGF21 expression in the liver, which is the main organ for circulating FGF21 production in humans and rodents (Fon Tacer et al., 2010). FGF21 has been shown to play a role in adipose tissue lipolysis, which increases the circulating FFAs that contribute to various pathologic conditions including hepatic steatosis. However, whether FGF21 plays a role in hypoxia-induced adipose tissue lipolysis is, as yet, unknown.

In the present study, we found that hypoxia increased systemic and hepatic expression of FGF21 as well as hormone-sensitive lipase-mediated adipose tissue lipolysis in mice. This hypoxia-induced lipolysis is attenuated in the *Fgf21* knockout (KO) mice. Furthermore, we found that the increase in FGF21 expression is not due to the activation of hypoxia-inducible factor (HIF) by hypoxia in hepatocytes, but through hypoxia-induced adipose tissue lipolysis leading to the mobilization of FFAs and the activation PPARα in the liver and hypoxia-induced oxidative stress.

## Material and Methods

### Cell Culture

HepG2 and H4IIE cells were purchased from the American Type Culture Collection (ATCC, Rockville, MD). *Hif-1α* and *Hif-2α* knockdown HepG2 cells were kindly provided by Dr. Bernhard Brune (Menrad et al., 2010). The cells were cultured in Dulbecco’s Modified Eagle Medium (DMEM), supplemented with 100 U/ml of penicillin, 100 µg/ml of streptomycin, and 10% fetal bovine serum (FBS), at 37°C in a humidified 5% CO_2_ environment. The culture media were changed every two days. The cells were subcultured after partial digestion with 0.25% trypsin-EDTA. In some experiments, HepG2 cells were treated with 1% O_2_ or chemical hypoxia inducer cobalt chloride (CoCl_2_, 0.01mg/µl) for 24 h. The PPARα antagonist, GW6471 (100 nmol/L), was used to determine whether FGF21 expression is mediated by PPARα. HIF-1α inhibitor, 2-Methoxyestradiol(2ME2) (100μm), was used for 12 h to determine whether the effects of hypoxia on FGF-21 are HIF regulated.

### Animal Studies

Male C57BL/6 mice were purchased from the Experimental Animal Center of Chongqing Medical University (Chongqing, China). *Fgf21* KO mice were kindly provided by Dr. Steve Kliewer (Potthoff et al., 2009). Animals were maintained under 12:12 h light:dark cycle conditions.

For CoCl_2_ treatment: 8–10-week old mice were treated with CoCl_2_ 6H_2_O (60 mg/kg, IP, Sigma) and fasted for 18 h to test. Experiment 1: Male C57BL/6 mice were divided randomly into three groups (Control, CoCl_2_, CoCl_2_+NAC). Experiment 2: Male wildtype (WT) and KO mice were divided randomly into four groups (WT, WT+CoCl_2_, KO, KO+CoCl_2_). Each group had 7 mice.

For intermittent hypoxia (IH) treatment: The murine model of IH exposure during sleep has been established and extensively utilized in the study of sleep apnea-associated morbidities. In this study, 8–10-week male C57BL/6 mice for exposure were divided into in two groups: an IH group and an intermittent air (IA) control group with 7 mice in each group. The IH paradigm consisted of 20.9% O_2_/8% O_2_ F_I_O_2_ alternate cycles (30 episodes/h) with 20 s at the nadir F_I_O_2_ for 12 h a day during daylight. Pulse oxyhemoglobin saturation (SpO_2_) changed in a recurrent manner with the nadir hemoglobin oxygen saturations mainly remaining in the range 60–70% to mimic hypoxia/reoxygenation events occurring in patients with moderate to severe obstructive sleep apnea. The IH exposure continued for eight weeks.

The humidity, ambient CO_2_, and temperature (22 ± 1°C) in the chambers were regularly monitored and maintained during the treatment. After IH/IA exposure, the mice were transferred to room air until they were humanely sacrificed for tissue collection. At the end of the experiment, the mice were anesthetized with Avertin (250 mg/kg), and tissue samples were collected for assays. Mice were treated according to the protocols reviewed and approved by the Institutional Animal Care and Use Committee of Chongqing University Three Gorges Hospital [2017(021)].

### Differentiation of 3T3-L1 Fibroblasts Into Adipocytes

3T3-L1 fibroblasts (ATCC, Rockville, MD) were cultured in DMEM that contained high glucose (4.5 g/L), glutamine (584 mg/L), and sodium pyruvate (110 mg/L) supplemented with 10% newborn calf serum (Gibco/Invitrogen; Carlsbad, CA, USA) and maintained at 37°C in 5% CO_2_. In accordance with a standard protocol, 3T3-L1 fibroblasts were differentiated into adipocytes. Briefly, differentiation was induced two days after confluency by the addition of DMEM containing 10% FBS and dexamethasone (1 µmol/L, Sigma-Aldrich; St. Louis, MO, USA), 3-isobutyl-1-methylxanthine (0.5 mmol/L, Sigma-Aldrich), and insulin (100 nmol/L) (Gibco/Invitrogen) for two days. The cells were then further incubated with DMEM supplemented with insulin for another two days. Fully differentiated adipocytes were maintained in DMEM at 37°C in a humidified atmosphere containing 5% CO_2_, and they were used 9–14 days after differentiation was initiated.

### Isolation and Culture of Primary Adipocytes

Male C57BL/6 mice were used to obtain primary adipocytes. Briefly, the mice were anesthetized and euthanized by cervical dislocation. The epididymal fat pads were harvested, washed in phosphate-buffered saline (PBS, pH 7.4) at room temperature, and minced thoroughly (2–3 mm) in collagenase solution (0.2 mg/ml of collagenase A, 4 ml/g of adipose tissue). This mixture was then incubated at 37°C and shaken at 120 rpm for 30 min. After digestion, the mixture was filtered through a 250-μm gauze mesh into a 50-ml conical polypropylene tube; it was then allowed to stand for 2–3 min. The floating layer of adipocytes was washed three times and incubated at 37°C in DMEM containing 1% bovine serum albumin.

### RNA Interference

siRNAs that target human HIF-1α and a negative mismatched control were designed and synthesized by Ambion, Inc. (Austin, TX). The siRNA sequences for HIF-1α were as follows: sense, GCUUAUUGAGAAUGAGGAGTT; antisense, CUCCUCAUUCUCAAUAAGCTC. After the monolayer cultures attained 50% confluence, the cells were transfected with 100 nmol/L of HIF-1α -targeted or negative-mismatched siRNA using Lipofectamine 2000 (Invitrogen) in accordance with the manufacturer’s instructions. Antibiotics were added to the medium 24 h after transfection, and the cells were used for the experimental procedures 48 h after transfection.

### Nuclear Extract Preparation

The cells were washed once on a dish with ice-cold PBS. Ice-cold buffer (10 mmol/L of Tris-HCl, pH 7.8, 1.5 mmol/L of MgCl2, and 10 mmol/L of KCl) containing freshly added 0.4 mmol/L of phenylmethylsulfonyl fluoride (PMSF), 0.5 mmol/L of dithiothreitol (DTT), and 1% protease inhibitor cocktail (Sigma-Aldrich) was overlaid on the cells in the dish and incubated for 10 min. Next, the cells were harvested by scraping with a rubber cell policeman and lysed by Dounce homogenization. The nuclei were pelleted by centrifugation and then resuspended in ice-cold buffer (20 mmol/L of Tris-HCl, pH 7.8, 420 mmol/L of KCl, 1.5 mmol/L of MgCl_2_, and 20% glycerol) containing freshly added 0.4 mmol/L of PMSF, 0.5 mmol/L of DTT, 1% protease inhibitor cocktail, and 1 mmol/L of Na_3_VO_4,_ and incubated for 30 min on ice with occasional tapping. The extracts were clarified by centrifugation at 12,000 g for 15 min at 4°C, placed in aliquots, and then stored at -80°C.

### Reactive Oxygen Species (ROS) Determination by Fluorescence Microscopy

The superoxide content in the HepG2 cells was examined by dihydroethidium (DHE, Molecular Probes, Eugene, OR) fluorescence microscopy. Nonfluorescent DHE is oxidized by superoxide, yielding a red fluorescent product, ethidium, that binds to nucleic acids, staining the nucleus a bright fluorescent red. Cryostat sections of the HepG2 cell chamber slides were incubated with 5 μmo/L of DHE for 30 min at 37°C in the dark. The ROS-catalyzed ethidium red fluorescence was examined using fluorescence microscopy.

### Quantitative Real-Time PCR

The mRNA expression was assessed by real-time PCR (RT-PCR). In brief, the total RNA (either from the frozen tissues or the cultured cells) was isolated with Trizol in accordance with the manufacturer’s protocol (Invitrogen) and reverse-transcribed using a GenAmp RNA PCR kit (Applied Biosystems, Foster City, CA). The cDNA was amplified in 96-well reaction plates with a SYBR green PCR Master Mix (Applied Biosystems) on an ABI 7500 RT-PCR thermocycler. **Table 1** lists the forward and reverse primers. The relative quantities of the target transcripts were calculated from the duplicate samples after normalization to a housekeeping gene, β-actin. After PCR amplification to confirm the specificity of the primers, dissociation curve analysis was performed. The relative mRNA expression was calculated using the 2^-△△CT^ method.

**Table 1:** Primer Sequence for RT-PCR.

Gene	Source	Sequences (Forward/Reverse 5’-3’)	
Glut1	Human	CATCAATGCCCCCCAGAA	AAGCGGCCCAGGATCAG
FGF21	Human	ACCTGGAGATCAGGGAGGAT	GCACAGGAACCTGGATGTCT
HIF-1α	Human	CCCAATGGATGATGACTTCC	TGGGTAGGAGATGGAGATGC
β-actin	Human	GAGACCTTCAACACCCC	ATAGCTCTTCTCCAGGGAGG
FGF21	Mouse	CCTCTAGGTTTCTTTGCCAACAG	AAGCTGCAGGCCTCAGGAT
β-actin	Mouse	GGCTGTATTCCCCTCCATCG	CCAGTTGGTAACAATGCCATGT

### Western Blot Analysis

Hepatic and adipose tissues were homogenized, and the HepG2 cells were lysed on ice for 30 min in RIPA buffer (50 mmol/L of Tris·HCl, pH 7.4, 150 mmol/L of NaCl, 2 mmol/L of EDTA, 4 mmol/L of Na_3_VO_4_, 40 mmol/L of NaF, 1% Triton X-100, 1 mmol/L PMSF, and 1% protease inhibitor cocktail) and centrifuged at 14,000 g for 10 min. The supernatants were collected. Aliquots containing 30 μg of protein were loaded onto 4–15% SDS-polyacrylamide gels. After electrophoresis, the proteins were transferred onto a polyvinylidene fluoride membrane. The membrane was probed with antibody against HIF-1α (BD, Sparks, MD), hormone-sensitive lipase (HSL) and p-HSL (Cell signaling, Danvers, MA), FGF21 (Abcam, San Francisco, CA), adipose triglyceride lipase (ATGL), protein kinase A (PKA) substrate (Cell Signaling technologies), and β-actin (Santa Cruz Biotechnologies, Santa Cruz, CA). Then, the membrane was processed with horseradish peroxidase-conjugated IgG. An enhanced chemiluminescence detection system (GE Healthcare, Piscataway, NJ) was used to envisage the protein bands, which were then quantified by densitometry analysis.

### Biochemical Assays

The FGF21 levels in the plasma and the HepG2 cell media were measured with an FGF21 ELISA kit (Biovendor, Modrice, Czech Republic) in accordance with the manufacturer’s instructions. The FFAs in the plasma and the 3T3-L1 adipocytes were measured using an NEFA C kit (Wako Chemicals, Richmond, VA). The glycerol in the plasma and the 3T3-L1 adipocytes culture supernatants were measured using a Glycerol Colorimetric Assay Kit (Cayman Chemical, Ann Arbor, MI). The plasma adrenaline and noradrenaline levels were measured using an ELISA kit from Labor Diagnostika Nord (Nordhorn, Germany). Cyclic AMP (cAMP) levels were measured in epididymal white adipose tissue (eWAT) using cAMP direct immunoassay kit (Abcam, Cambridge, MA) according to the manufacturer’s protocol.

### Statistical Analysis

Data are expressed as means ± SEM. Statistical analysis was performed using GraphPad Prism 8 (GraphPad Software Inc., San Diego, CA, USA). Statistical comparisons were made using two -way analysis of variance (ANOVA) with Bonferroni’s *post hoc* test or one-way ANOVA with Tukey’s *post hoc* test or Student *t*-test where it was appropriate. Differences were considered to be significant at *P*<0.05. Statistical methods and corresponding *p* values for data shown in each panel were included in figure legends.

## Results

### Hypoxia Increases FGF21 Expression in Mice

**Figure 1:**
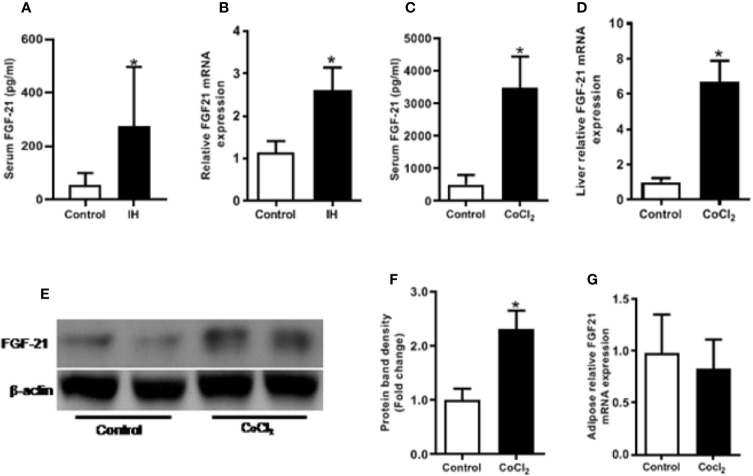
Hypoxia increases FGF21 expression in mice. Control vs IH (intermittent hypoxia), Male C57BL/6 mice were divided into two groups and exposed for eight weeks to either intermittent hypoxia or intermittent air (control group). The serum FGF21 level was measured by ELISA in IH **(A)**. Hepatic *Fgf21* mRNA expression was measured by RT-PCR **(B)**. Control vs CoCl_2_, The mice were also exposed to the chemical hypoxia mimic, CoCl_2_, and fasted for 18 h. The serum FGF21 level was measured by ELISA **(C)**. Liver *Fgf21* mRNA **(D)** and protein **(E, F)** expression were measured by RT-PCR and Western blot, respectively. WAT *Fgf*21 mRNA **(G)** was measured by RT-PCR. *P < 0.05.

IH treatment caused a significant (p < 0.05) increase in the circulating FGF21 level (**Figure 1A**), and hepatic *Fgf21* mRNA level (**Figure 1B**). Chemical hypoxia treatment increased the circulating FGF21 levels, the hepatic *Fgf21* mRNA and protein expression by eight folds, six folds and two folds, respectively, compared with the control mice (**Figures 1C–F**). However, chemical hypoxia treatment did not change the *Fgf21* mRNA expression in the WAT (**Figure 1G**).

### Hypoxia-Induced Change in FGF21 Expression Is Not Mediated by HIF

**Figure 2:**
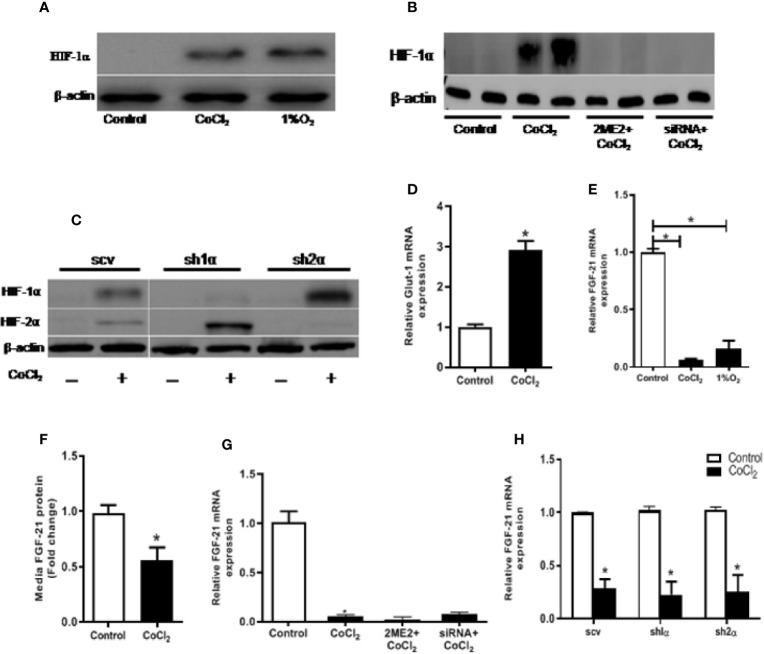
Hypoxia-induced change in FGF21 expression is not mediated by HIF in HepG2 cells. HepG2 cells were treated with CoCl_2_ or exposed to 1% O_2_ in the absence of serum for 18 h. HIF-1α protein expressions were determined by Western blot **(A)**. Expression of HIF-1α and HIF-2α protein were measured by Western blot **(C)**. *Glut-1* mRNA levels were determined by RT-PCR **(D)**. Cellular *Fgf21* mRNA **(E)** and secreted protein in the median **(F)** were measured by RT-PCR and ELISA, respectively. HepG2 cells were treated with the HIF-1α inhibitor, 2ME2 (100 µM), for 12 h or transfected with HIF-1α siRNA for 48 h, respectively, followed by CoCl_2_ treatment for 18 h. Expressions of HIF-1α protein **(B)** and *Fgf21* mRNA **(G)** were measured. WT HepG2 (scv), *Hif-1α* KO (sh1α), and *Hif-2α* KO (sh2α) HepG2 cells were exposed to CoCl_2_ in the absence of serum for 18 h. *Fgf21* mRNA expression was measured by RT-PCR **(H)**. *P < 0.05.

The immediate cellular response to hypoxia is the accumulation of the HIF-α protein. As shown in **Figure 2A**, both physical and chemical hypoxia induced a considerable elevation in HIF-1α protein levels. Furthermore, we examined the expression of GLUT-1, a well-known HIF-1α transcription target, in response to the hypoxia treatment. As expected, hypoxia significantly increased *Glut*-1 mRNA expression in the HepG2 cells (**Figure 2D**). However, unlike the *in vivo* study, hypoxia treatment did not increase, rather decreased the cellular *Fgf21* mRNA expression and protein secretion in the HepG2 cells (**Figures 2E, F**). These results suggest that FGF21 expression is differentially regulated in hepatocytes and in animal.

We treated the HepG2 cells with 2-ME2 or *Hif-1α* specific siRNA to determine whether the change in FGF21 expression was mediated by the HIF protein level. Both of these treatments decreased the nuclear HIF-1α protein levels (**Figure 2B**). However, reducing HIF-1α did not affect the CoCl_2_-induced reduction of *Fgf21* mRNA expression **(****Figure 2G**). We further investigated the correlation between FGF21 expression and HIF levels using HepG2-derived stable cell lines, in which the *Hif-1α* (sh1α) or *Hif-2α* (sh2α) was deleted, respectively. CoCl_2_ treatment did not increase the HIF-1α in HepG2 cells, but significantly increased the HIF-2α expression when the *Hif-1α* was genetically deleted (**Figure 2C**). In a similar fashion, deletion of the *Hif-2α* resulted in insensitizing of the HIF-2α protein to CoCl_2_ treatment, but significantly increased the HIF-1α protein (**Figure 2C**). Chemical hypoxia did not change the FGF21 expression in either *Hif-1α*- or *Hif-2α*-deficient cell lines compared with the control (**Figure 2H**). In summary, CoCl_2_-induced reduction in FGF21 expression in hepatocytes is independent of the HIF-α protein level.

### The Hypoxia-Induced FGF21 Down-Regulation in HepG2 Cells and Up-Regulation in Mice Are Dependent on Oxidative Stress

**Figure 3:**
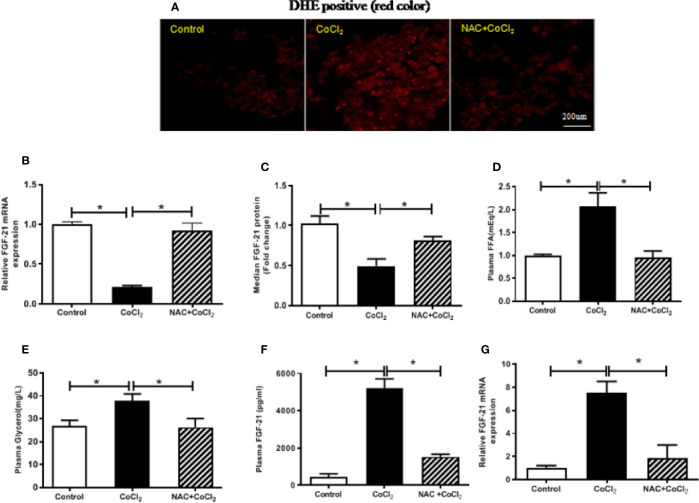
The hypoxia-induced FGF21 down-regulation in the HepG2 cells and up-regulation in the mice are dependent on oxidative stress. The HepG2 cells were exposed to CoCl_2_ with/without NAC in the absence of serum for 18 h. The mice were treated with CoCl_2_ with/without NAC and fasted for 18 h. ROS accumulation in the HepG2 cells was examined by DHE staining (red) **(A)**. Cellular *Fgf21* mRNA **(B)** and secreted FGF21 protein in the media **(C)** of HepG2 cells were measured by RT-PCR and ELISA, respectively. Plasma FFA **(D)** and glycerol **(E)** levels of the mice were measured. The plasma FGF21 level **(F)** and liver *Fgf21* mRNA expression **(G)** of the mice were measured by ELISA and RT-PCR, respectively. *P < 0.05.

Previous studies have shown that ROS generation is induced by hypoxia (Siques et al., 2018). A potent antioxidant, NAC, together with CoCl_2,_ was used to treat the HepG2 cells to determine whether the reduction of FGF21 expression in the HepG2 cells was due to CoCl_2_-induced oxidative stress. CoCl_2_ treatment induced a significant increase in the production of superoxide, which was evaluated by DHE staining. Also, administration of NAC significantly inhibited the CoCl_2_-induced ROS accumulation in HepG2 cells (**Figure 3A**). It is important to note that the CoCl_2_-induced decrease of cellular *Fgf21* mRNA was evidently reduced by the NAC treatment (**Figure 3B**). Similarly, FGF21 secretion was also restored (**Figure 3C**). These results suggest that alteration of FGF21 expression in hepatocytes by hypoxia is inhibited by hypoxia-induced oxidative stress.

In the similar way, the mice were treated with CoCl_2_ in the presence of NAC to determine whether the increased FGF21 expression was due to hypoxia-induced oxidative stress. Hypoxia-increased plasma FFA and glycerol were decreased by NAC treatment (**Figures 3D, E**), indicating that hypoxia-induced oxidative stress is probably the main cause of adipose tissue lipolysis. It is important to note that chemical hypoxia-induced elevation of FGF21 protein in the plasma and mRNA expression in the liver were inhibited by NAC treatment (**Figures 3F, G**), suggesting that the increase of FGF21 expression in the plasma and liver of hypoxia-treated mice is regulated by oxidative stress.

### Hypoxia Induces Adipose Lipolysis

**Figure 4:**
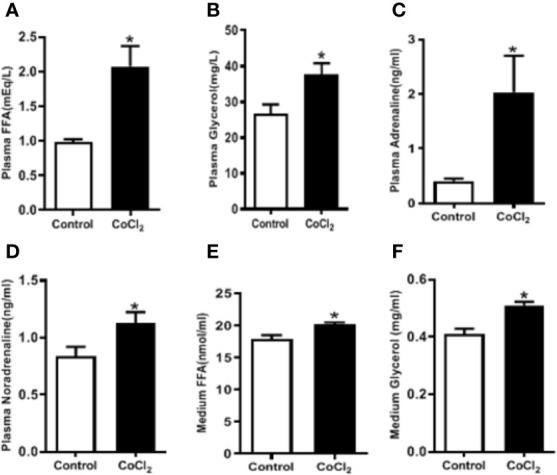
Hypoxia induces adipose lipolysis. The mice were treated with CoCl_2_ and fasted for 18 h. Plasma FFA **(A)**, glycerol **(B)**, adrenaline **(C)**, and noradrenaline **(D)** levels were measured by ELISA, respectively. The 3T3-L1 adipocytes were exposed to CoCl_2_ in the absence of serum for 18 h. Median FFA **(E)** and glycerol **(F)** levels were measured by ELISA. *P < 0.05.

Adipose tissue lipolysis-released FFAs are ligands of PPARα, which is a major transcription factor that regulates the expression of *Fgf21* in the liver. To investigate whether CoCl_2_-induced FGF21 up-regulation in mice is mediated by the increase of adipose tissue lipolysis, we measured the plasma FFA and glycerol levels. CoCl_2_ treatment increased plasma FFA level by two folds, and the glycerol level by one and a half folds (**Figures 4A, B**); indicating an increase in lipolysis under hypoxic conditions. Additionally, CoCl_2_ treatment significantly reduced mouse eWAT weight (0.35 ± 0.01 g vs. 0.49 ± 0.03 g). To further investigate whether hypoxia-induced adipose lipolysis is *via* a systemic effect on catecholamine release, the plasma adrenaline and noradrenaline were measured. As shown in **Figures 4C, D**, the hypoxia treatment significantly increased the plasma level of adrenaline by four folds and moderately increased the level of noradrenaline. Adipose tissue is the main location for excess fat storage and the main source of FFA mobilization during fasting and other metabolic states in which it is needed. We then investigated whether hypoxia increases FFA release from adipocytes. The 3T3L1 adipocytes were treated with CoCl_2_ for 18 h. This led to an adipocyte lipolysis assessed by FFA and glycerol concentrations in the media (**Figures 4E, F**). A systemic increase in FFA could stimulate FGF21 expression in the liver through PPARα activation. Indeed, linoleate-induced FGF21 expression was partially inhibited by a PPARα antagonist, GW6471, in the HepG2 and H4IIE cells (**Supplementary Figure S1**).

### *Fgf21* KO Inhibits Hypoxia-Induced Adipose Tissue Lipolysis

**Figure 5:**
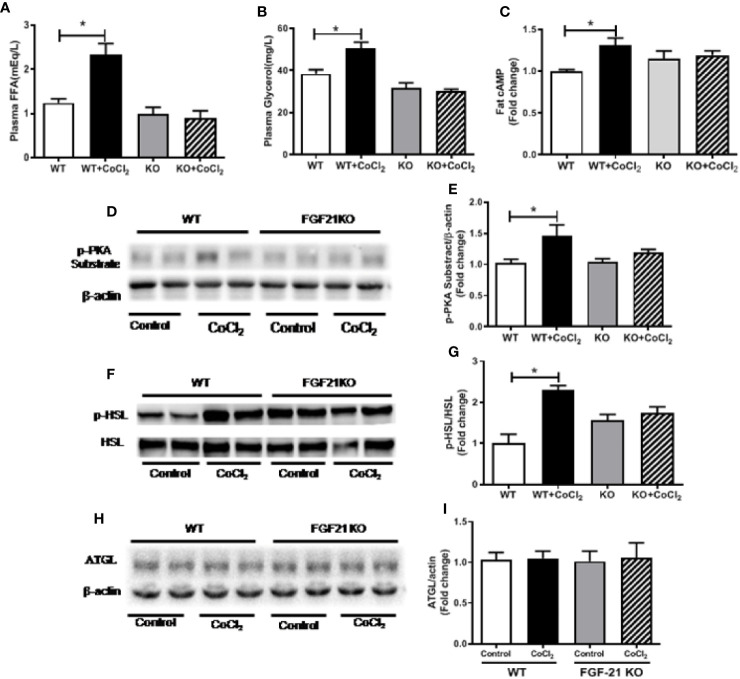
*Fgf21* KO inhibits CoCl_2_-induced adipose tissue lipolysis. WT and *Fgf21* KO mice were treated with CoCl_2_ and fasted for 18 h. Plasma FFA **(A)** and glycerol **(B)** levels were measured. Adipose cAMP **(C)** levels were measured by immunoassay kit. Adipose protein kinase A (PKA) **(D–E)**, p-HSL/HSL **(F–G)**, and adipose triglyceride lipase (ATGL) **(H–I)** levels were measured by Western blot. *P < 0.05.

**Figure 6:**
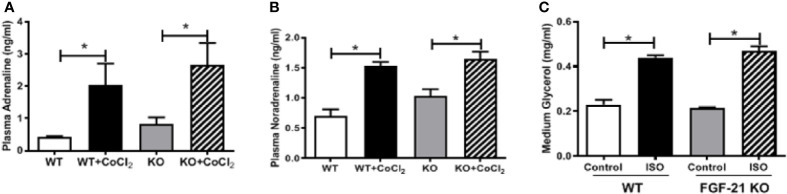
FGF21 regulation of lipolysis is independent of catecholamine action. WT and *Fgf21* KO mice were treated with CoCl_2_ and fasted for 18 h. Plasma adrenaline **(A)** and noradrenaline **(B)** levels were measured by ELISA. Primary adipocytes isolated from WT and *Fgf21* KO mice were treated with isoproterenol (ISO, 1 mM) for 2 h. Median glycerol was measured **(C)**. *P < 0.05.

An important function of FGF21 is to regulate adipose lipolysis. The *Fgf21* KO mice were treated by hypoxia as described above to investigate whether FGF21 plays a role in the lipolysis of adipose tissue under hypoxic conditions. No observable effect was detected on the overall behavior of the mice in response to the CoCl_2_ treatment when FGF21 was deleted. Surprisingly, the *Fgf21* KO mice were found to be resistant to hypoxia-induced adipose lipolysis, as shown by the lack of increase in plasma FFA and glycerol concentrations (**Figures 5A, B**), indicating that FGF21 plays a critical role in hypoxia-induced adipose lipolysis. Furthermore, hypoxia significantly increased adipose cAMP levels and induced phosphorylation of PKA and HSL in WT mice, but not in the *Fgf21* KO mice (**Figures 5C–G**), whereas another major enzyme, responsible for TG breakdown in adipose tissue, ATGL, was unchanged (**Figures 5H, I**). The catecholamine-mediated adrenergic response is an important mechanism in adipose tissue lipolysis (Koppo et al., 2012). The plasma levels of adrenaline and noradrenaline were measured. As shown, hypoxia treatment increased the catecholamine levels in both WT and *Fgf21* KO mice (**Figures 6A, B**). We treated primary adipocytes isolated from the WT and *Fgf21* KO mice with synthetic catecholamine, isoproterenol (1 mmol/L), for 2 h to determine whether catecholamine-induced adipose lipolysis is mediated by FGF21. The glycerol secreted from the primary adipocytes was significantly increased by isoproterenol treatment in both WT and *Fgf21* KO mice (**Figure 6C**), suggesting that regulation of lipolysis by FGF21 is independent of any catecholamine action.

## Discussion

Previous studies have shown that lipid dysregulation is associated with the tissue hypoxia induced by many pathologic conditions such as cancer (Koizume et al., 2015; Deep and Schlaepfer, 2016), non-alcoholic fatty liver disease (Chen et al., 2019), and cardiovascular disease (Mylonis et al., 2019). FGF21 is a newly described hepatokine that is important in lipid homeostasis. However, the potential link between hypoxia-induced lipolysis and FGF21 expression is not yet investigated. Therefore, we sought to determine how FGF21 is regulated under hypoxic conditions *in vivo* and *in vitro*, respectively.

In the present study, we demonstrated that hypoxia increases FGF21 expression in mice (**Figures 1A–F**). Increased FGF21 expression by hypoxia is not due to the activation of HIF in hepatocytes (**Figure 2H**), but rather through hypoxia-induced adipose tissue lipolysis (**Figures 4A, B**), which leads to the mobilization of FFAs and the activation FGF21 synthesis in the liver through PPARα activation (**Supplementary Figure S1**). We further demonstrated that FGF21 is required for adipose tissue lipolysis (**Figures 5A–G**).

An important finding of this study is that hypoxia induces a dramatic increase in FGF21 expression in circulation (**Figures 1A–C**) and hepatic tissue (**Figures 1D–F**), but not in WAT (**Figure 1G**). Previous study has demonstrated that circulating FGF21 was mainly produced from liver, as circulating FGF21 level was completely abolished in liver-specific *Fgf21* KO mice, but not in adipose-specific *Fgf21* KO mice (Markan et al., 2014). It is clear that adipose tissue also expresses FGF21 (Dutchak et al., 2012). However, adipose-derived FGF21 acts locally to modulate PPARγ activity in autocrine or paracrine manner involving in the browning of white adipose tissue (Dutchak et al., 2012; Fisher et al., 2012) and is not associated with lipolysis (Arner et al., 2008; Li et al., 2009). Although hepatocytes are a major type of FGF21-producing cells, our data showed that the hypoxia-induced FGF21 elevation in the plasma and hepatic tissue is not related to direct exposure of hepatocytes to hypoxia (**Figures 2E, F** and **3B, C**). It is well-known that hepatic PPARα activation by FFAs upregulates FGF21 expression (Cuevas-Ramos et al., 2012). Our results showed that hypoxia exposure increased FFA release in 3T3-L1 adipocytes (**Figures 4E, F**). Therefore, the hypoxia-induced rise in FGF21 expression in circulation and hepatic tissue is likely mediated by increased levels of FFAs from adipose tissue lipolysis.

Adipose tissue lipolysis and adipogenesis are exquisitely controlled processes. It is widely recognized that cAMP signaling *via* PKA is important both in adipogenesis and in lipolysis in WAT (Rogne and Tasken, 2014). In addition to catecholamines, several other non-adrenergic lipolysis factors such as parathyroid hormone (Larsson et al., 2016), cardiac natriuretic peptide (Collins, 2014), thyroid-stimulating hormone (Endo and Kobayashi, 2012), growth hormone (Yip and Goodman, 1999), can also regulate lipolytic process in adipose tissue. On the other hand, insulin signaling pathway, including phosphatidylinositol 3-kinase, phosphoinositide- dependent kinase, and protein kinase B (Akt) (Whitman et al., 1988; Stokoe et al., 1997), plays a major role in antilipolytic process (Jensen and Nielsen, 2007). Akt further activates phosphodiesterase 3B, which mediates degradation of cAMP to 5′-AMP and attenuates PKA-dependent stimulation of lipolysis process (Choi et al., 2006). Our previous study demonstrated that alcohol exposure-induced FGF21 expression stimulated adipose lipolysis through systemic catecholamine reaction (Zhao et al., 2015). Consistent with this finding, our current results showed that hypoxia increased β-AR-cAMP-PKA-HSL pathway (**Figures 5A–G**). We also shown in the **Figures 4E, F** that CoCl_2_ treatment moderately increased media FFA and glycerol concentration in 3T3L1 adipocytes, indicating that the basal lipolysis (independent of catecholamine-induced) was increased by CoCl_2_. However, the extend of the increase (FFA, ~15%) was much smaller compared to the total lipolysis in animal (including catecholamine-induced) by CoCl_2_ (FFA~100%, **Figure 4A**). These data suggest that CoCl_2_ induce lipolysis mainly attributes to the systemic activation of catecholamine signaling, which may involves FGF21 activation. Thus, the elevation of FGF21 by hypoxia is a result of hypoxia-induced activation of the adrenergic receptor response, leading to an increase in lipolysis and FFA release in the adipose tissue.

Our results showed that hypoxia-induced FGF21 expression is differentially regulated in intact animals and in hepatocytes (**Figures 1A-F**, **2E, F**). In animals, hypoxia leads to adipose lipolysis, which in turn increases FFA concentration in the circulation, subsequently activating PPARα in the liver. However, hypoxia inhibits PPARα protein expression through HIF-1α and HIF-2α accumulation (Narravula and Colgan, 2001). The net effect of the induction of FGF21 by hypoxia is therefore a balance between FFA-induced transcriptional activation and HIF-induced down-regulation of PPARα. By contrast, *in vitro* hepatocyte culture is not subject to systemic lipolysis, and thus, the HIF-repressed PPARα contributed to the FGF21 expression. Interestingly, anti-oxidant (NAC) treatment inhibited the changes in FGF21 expression both *in vivo* (increase) and *in vitro* (decrease) (**Figures 3B–G**). NAC has been used widely for its anti-oxidant effect in cell culture and in animal experiments. As shown in our previous study (Liu et al., 2012), NAC itself did not induce changes in FGF21 expression. Our study clearly showed that NAC markedly inhibited CoCl_2_-suppressed FGF21 expression in HepG2 cell and CoCl_2_-increased FGF21 expression in mice, indicating the role of oxidative stress response. Therefore, it is likely that hypoxia-induced oxidative stress is responsible for the decrease in FGF21 expression due to the inactivation of PPARa in hepatocytes, whereas the oxidative stress may increase global catecholamine concentration and enhance adipose tissue lipolysis, subsequently increasing FGF21 expression in animals.

It is well-known that HIF-α regulates the expression of numerous genes in response to hypoxia. However, the hypoxia-responsive element has not yet been found in the promoter region of FGF21. In this study, it was found that inhibition of HIF-α by its specific inhibitor and siRNA, or by genetic deletion of *Hif-1α* or *Hif-2α*, decreased the CoCl_2_-induced HIF-α protein but did not affect the CoCl_2_-decreased *Fgf21* mRNA level (**Figures 2B, C, G, H**), indicating that the regulation of FGF21 by hypoxia may be independent of HIF-α transcriptional activity. In summary, our results suggest that HIF is not involved in hypoxia regulation of FGF21 expression in hepatocytes.

What is the physiological significance of the link between hypoxia and FGF21 elevation? The finding that FFA released from adipose tissue mediates hypoxia-induced FGF21 expression may explain the circadian rhythm of FFAs and FGF21 in patients with sleep apnea. In humans, both FFA and FGF21 levels peak at midnight during deep sleep and are at their lowest at noon (Moller et al., 1990; Yu et al., 2011). A recent study has demonstrated that patients with severe sleep apnea exhibit a marked and rapid increase in FFAs compared with control subjects after sleep onset, and that attenuation of the hypoxia *via* supplementation of oxygen normalizes the FFA profile (Jun et al., 2011). This strongly suggests that hypoxia may account for the FFA release in patients with sleep disorders. However, the nocturnal rise of FGF21 is secondary to the midnight increase of FFA in humans. Thus, the hypoxia status caused by severe sleep apnea after sleep onset results in an increase of circulating FFA, which may contribute to the nocturnal rise in FGF21. The hypoxia-induced increase of FFAs and glycerol in the blood (**Figures 5A, B**) and cAMP-PKA-HSL pathway in the adipose tissue were completely blocked (**Figures 5C–G**) in *Fgf21* KO mice, evidently suggesting that FGF21 is required for hypoxia-induced lipolysis. However, it seems that endogenous FGF21 is barely involved in catecholamine-induced adipocyte lipolysis since *Fgf21* KO mice have similar responses to isoproterenol for lipolysis compared with WT mice.

The role of endogenous FGF21 favoring lipolysis in adipose tissue under hypoxic insult may explain the rise in circulating FFA levels in obese subjects. Obesity induces a hypoxic state in adipocytes. In addition to inflammation, hypoxia enhances adipocyte lipolysis, and releases lipolytic products including FFAs, which in turn enhance hepatic FGF21 production through the activation of PPARα. This feed forward effect increases obesity-induced circulating FFA concentration, leading to insulin resistance. By contrast, FGF21 reduces hormone-induced lipolysis through a negative feedback loop (Chen et al., 2011). The differential feedback regulation of adipocyte lipolysis by FGF21 in differing metabolic states may be indicative of a complex fine-tuning mechanism in lipid homeostasis. The regulation of lipolytic activity by FGF21 under distinct physiological and pathological conditions thus remains to be clarified.

In summary, the present study demonstrated that hypoxia induces adipocyte lipolysis leading to an increased FGF21 expression in mice. Hypoxia stimulates adipocyte lipolysis by oxidative stress-mediated elevation in the catecholamine response and enhances HSL phosphorylation; this process requires FGF21. The positive feedback regulation mechanism between FGF21 and adipocyte lipolysis in hypoxia indicates that FGF21 regulates lipolysis in response to varying metabolic states. As an adaptive response to hypoxia, HIF-α protein is increased in the liver but is not possibly involved in the hepatic expression of FGF21. These findings provide new insight into the molecular mechanism underlying the regulation of FGF21 under hypoxic conditions.

## Data Availability Statement

The raw data supporting the conclusions of this article will be made available by the authors, without undue reservation, to any qualified researcher.

## Ethics Statement

The animal study was reviewed and approved by the Institutional Animal Care and Use Committee of Chongqing University Three Gorges Hospital.

## Author Contributions

GW, YL, and CT designed the study and wrote the manuscript, GW and YL performed the experiments, analyzed, and interpreted the data. WF contributed to the critical discussion and manuscript revision. XA and WL assisted in the completion of the project. All authors contributed to the article and approved the submitted version.

## Funding

This work was supported by National Natural Science Foundation of China (81873571, 81300311), Chongqing Natural Science Foundation (cstc2019jcyj-msxmX0774), Projects of Medical and Health Technology Program in Zhejiang Province (2015KYB234, 2017KY720), and Wenzhou Science and Technology Bureau Foundation (ZS2017016, Y20140739, Y20150094).

## Conflict of Interest

The authors declare that the research was conducted in the absence of any commercial or financial relationships that could be construed as a potential conflict of interest.

## Supplementary Material

Supplementary Figure 1 FFA-induced FGF21 up-regulation is mediated by PPARα. HepG2 **(A)** and H4IIE **(B)** cells were treated with linoleate (150 μmol/L) and a PPARα antagonist, GW6471 (100 nmol/L), for 24 hours. Cellular *Fgf21* mRNA levels were measured by RT-PCR. *P < 0.05.
