# Supplementary figures and images for: Hypoxia-Induced Adipose Lipolysis Requires Fibroblast Growth Factor 21
Source: Front Pharmacol. 2020 Aug 14;11:1279. doi: 10.3389/fphar.2020.01279 (PMC7456904; doi:10.3389/fphar.2020.01279)

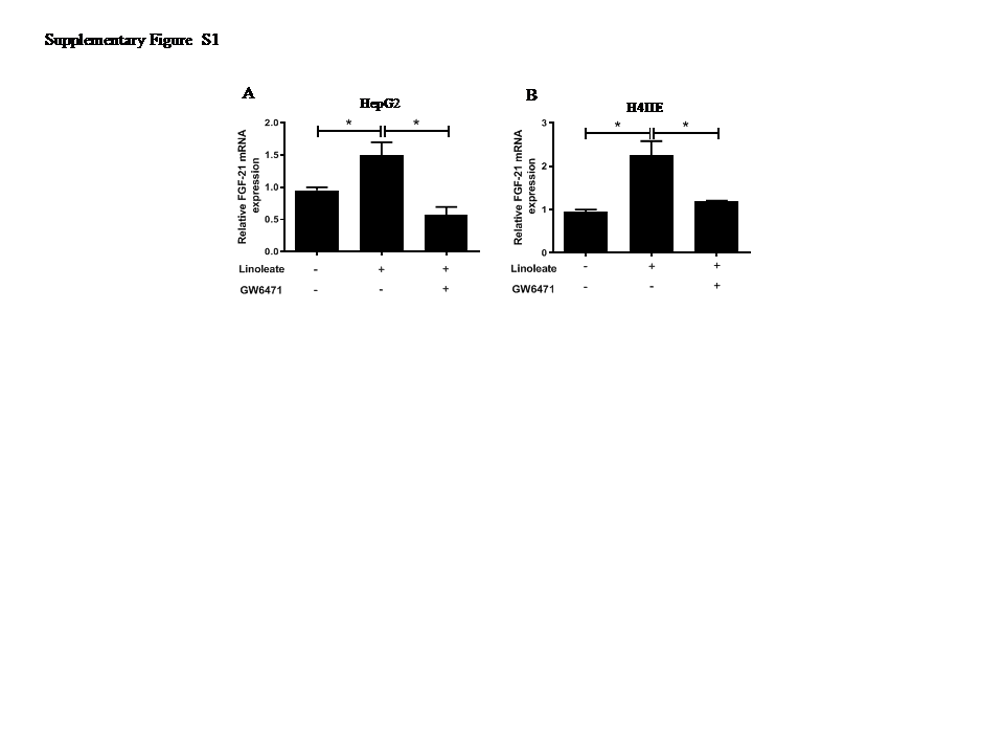

Supplement: Supplementary Figure 1 — FFA-induced FGF21 up-regulation is mediated by PPARα. HepG2 (A) and H4IIE (B) cells were treated with linoleate (150 μmol/L) and a PPARα antagonist, GW6471 (100 nmol/L), for 24 hours. Cellular Fgf21 mRNA levels were measured by RT-PCR. *P < 0.05. [file Image_1.tif]
